# Assessing the Impact and Suitability of Dense Carbon Dioxide as a Green Solvent for the Treatment of PMMA of Historical Value

**DOI:** 10.3390/polym15030566

**Published:** 2023-01-21

**Authors:** Angelica Bartoletti, Inês Soares, Ana Maria Ramos, Yvonne Shashoua, Anita Quye, Teresa Casimiro, Joana Lia Ferreira

**Affiliations:** 1 LAQV-REQUIMTE and Department of Conservation and Restoration, NOVA School of Science and Technology, Universidade NOVA de Lisboa, 2829-516 Caparica, Portugal; 2 LAQV-REQUIMTE and Department of Chemistry, NOVA School of Science and Technology, Universidade NOVA de Lisboa, 2829-516 Caparica, Portugal; 3 Environmental Archaeology and Materials Science, National Museum of Denmark, 2800 Kongens Lyngby, Denmark; 4 Kelvin Centre for Conservation and Cultural Heritage Research, School of Culture and Creative Arts, University of Glasgow, Glasgow G12 8QH, UK; 5 Centro Interuniversitário de História das Ciências e da Tecnologia, Department of Conservation and Restoration, NOVA School of Science and Technology, Universidade NOVA de Lisboa, 2829-516 Caparica, Portugal

**Keywords:** plastics, museum and design objects, poly(methyl methacrylate) (PMMA), conservation, sustainable conservation, supercritical carbon dioxide

## Abstract

Surface cleaning of plastic materials of historical value can be challenging due to the high risk of inducing detrimental effects and visual alterations. As a result, recent studies have focused on researching new approaches that might reduce the associated hazards and, at the same time, minimize the environmental impact by employing biodegradable and green materials. In this context, the present work investigates the effects and potential suitability of dense carbon dioxide (CO_2_) as an alternative and green solvent for cleaning plastic materials of historical value. The results of extensive trials with CO_2_ in different phases (supercritical, liquid, and vapor) and under various conditions (pressure, temperature, exposure, and depressurization time) are reported for new, transparent, thick poly(methyl methacrylate) (PMMA) samples. The impact of CO_2_ on the weight, the appearance of the samples (dimensions, color, gloss, and surface texture), and modifications to their physicochemical and mechanical properties were monitored via a multi-analytical approach that included optical microscopy, Raman and attenuated total reflection Fourier transform infrared (ATR-FTIR) spectroscopies, and micro-indentation (Vickers hardness). Results showed that CO_2_ induced undesirable and irreversible changes in PMMA samples (i.e., formation of fractures and stress-induced cracking, drastic decrease in the surface hardness of the samples), independent of the conditions used (i.e., temperature, pressure, CO_2_ phase, and exposure time).

## 1. Introduction

Over the last 30 years, the challenge of conserving plastic materials has come to the forefront of heritage science. Despite being constantly demonized as a threat to the environment and wildlife because it is long-lasting and difficult to decompose, dispose of, or recycle, plastic is among the most fragile materials that can be encountered in museums and other heritage collections [1,2,3]. Signs of degradation can become readily visible within a few decades [2] as discoloration, yellowing, deformation, cracks or crazing on surfaces, blooming or weeping of additives that migrate to the surface, formation of superficial degradation products, embrittlement, or even total disintegration of the object, as a result of plasticizer loss or cross-linking [2,4].

Surface cleaning is an important factor in the care of plastic artifacts to increase their material stability while improving or restoring their visual appearance. However, conservation treatments of plastic objects and works of art, such as cleaning or consolidation, still pose considerable challenges to conservators, who must adhere to the ethical principles of reversibility and minimal damage [5]. Given the vast diversity of plastics, it is complex to outline common guidelines, and some types of plastics are more difficult to treat compared with others, such as poly(methyl methacrylate) (PMMA), which is highly prone to scratching [6,7,8]. While studies have mainly focused on understanding degradation pathways [9,10,11,12], developing rapid and in situ methods for plastics identification and characterization [13,14,15,16,17], and designing preventive conservation strategies [18,19,20,21,22,23], knowledge and expertise surrounding interventive conservation approaches (i.e., cleaning and consolidation) remain limited.

The research project POPART (Preservation of Plastic Artefacts in Museum Collections, 2008–2012) was the first to perform an extensive evaluation of cleaning options for various plastics, investigating dry, aqueous, and nonaqueous techniques and their effectiveness at removing sebum and carbonaceous soils [8]. The outcomes from this project suggested that satisfactory results can be achieved by using a polyester microfiber cloth dampened with anionic and nonionic detergent solutions, but with the potential drawback of inducing a few scratches and leaving cleaning residues. PMMA, which among plastics is considered the most stable [9,24], was found to be susceptible to scratching.

After the POPART project, research into developing new cleaning options for plastics remains relatively sparse. Recent studies have explored the use of more sustainable conservation approaches, such as deep eutectic solvent (DES) formulations for the removal of degraded gelatin on cellulose nitrate cinematographic films [25]; natural and biodegradable solvents, such as limonene and ethyl lactate; nonionic surfactant based on alkoxylated fatty alcohols (commercial product Plurafac^®^ LF 900) [26]; and various types of confining systems and gels for the cleaning of PMMA and other plastics [27,28,29].

Research by Kampasakali et al. found that limonene (applied with an Evolon^®^ CR cloth) was effective at cleaning plastics as it satisfactorily removed both the carbon and sebum soil types. However, solvent residues were noted on the surface after cleaning and required further clearance with a dry cloth. Plurafac^®^ LF 900 proved to be very effective in removing carbonaceous soil, while ethyl lactate removed sebum soil, but left stains of soil mixed with solvent residues on the surface [26]. Confining systems and gels, such as agar, gellan gum, Nanorestore Gel MWR, or Nanorestore Gel Peggy 5 or 6, were shown to reduce mechanical friction and, hence, induce significantly less damage compared with traditional cleaning tools. In terms of cleaning efficacy, however, the gels did not perform particularly well, especially in removing sebum soil [28,29]. While these studies have undoubtedly expanded the range of suitable options available to conservators, definitive and safer solutions for cleaning the most susceptible plastics are still needed. In addition, interest is growing in sustainable chemical processes and supercritical fluid technology, particularly carbon dioxide.

Supercritical carbon dioxide (scCO_2_) is considered a green solvent. It is chemically stable, relatively inert, nontoxic, and nonflammable. It is abundant in nature, but is also available as a by-product of many industrial processes and can be readily recycled [30,31]. scCO_2_ is widely used as an alternative to organic solvents in various chemical processes, including the synthesis of polymers [32,33], preparation of pharmaceutical formulations or drug release [34,35], extraction of essential oils [36,37,38] or caffeine from coffee beans [39,40], industrial and precision cleaning, and decontamination [41,42,43,44,45].

Carbon dioxide (CO_2_) naturally occurs as a gas in the atmosphere, but under certain conditions of pressure and temperature, it also acts as a solid (known as dry ice or snow), liquid, and supercritical fluid, exhibiting unique and versatile features. A supercritical fluid is a substance that, above its critical temperature and pressure, presents simultaneously physicochemical properties between those of a gas and a liquid (i.e., density, solvation power, viscosity, and diffusivity) [31,46]. Supercritical carbon dioxide exhibits a readily accessible critical point (T_c_ = 31 °C and p_c_ = 7.38 MPa) compared with other compounds, such as water (T_c_ = 373 °C and p_c_ = 22 MPa) [47].

The most significant advantage of working with scCO_2_ is its tunability. Minimal variations in pressure and/or temperature conditions can lead to significant modifications of its phase and properties. For example, after use, it can be easily released as a gas simply by returning to atmospheric pressure and temperature without leaving residues. Supercritical CO_2_ is a good solvent for nonpolar and very slightly polar compounds. However, its solvation power is directly proportional to its density and, hence, to the applied pressure. In addition, various separating agents (i.e., solvents, surfactants, etc.) can be used to increase or decrease its polarity, such as ethanol or methanol [46]. These unique and versatile features make scCO_2_ a perfect candidate for application in the heritage conservation field. Despite its very limited use in this field, liquid and supercritical CO_2_ have been tested in various treatments on a range of different materials, such as drying of waterlogged wood [48,49]; removal of various pesticides from objects in ethnographic collections comprising wood, leather, and textiles [50,51]; deacidification of paper-based objects [52,53,54,55,56,57]; and cleaning and disinfection of paper [58] and textiles [59,60,61,62,63].

The use of liquid/supercritical CO_2_ showed potential for cleaning fragile materials. Sousa et al. [59] tested liquid/supercritical CO_2_ on an extensively deteriorated silk textile from the 18th century that disintegrated on handling. Trials demonstrated that CO_2_ did not induce physical damage or promote material loss and enabled satisfactory soil removal, which could not be achieved using traditional approaches.

The use of CO_2_ remains relatively unexplored in treating plastics. Supercritical CO_2_ was applied for the first and only time on various plastics and cellulose acetate textiles during the POPART project, and trials led to unsatisfactory results. However, it should be noted that tests were limited and were performed under undisclosed conditions using an industrial apparatus, with the test plastics packed inside nylon stockings [8].

Considering the potential benefits of CO_2_ for cleaning applications (i.e., no or minimal interaction with the substrate, no residues left after treatment, and the possibility of easily fine-tuning the solvation strength), the present work aimed at reassessing the suitability of CO_2_ for application on plastics. PMMA was selected for this study, as it is one of the most common plastics found in post-1945 artworks, and despite being considered a stable plastic compared with other types, it can be easily damaged and scratched with consequent loss of original gloss. Extensive trials were performed on new PMMA samples using liquid and supercritical CO_2_, exploring a wide range of experimental conditions (i.e., temperature, pressure, and exposure time) and using a multianalytical characterization approach to monitor potential changes in the samples. Vapor CO_2_ was used as a comparative cleaning agent. The ultimate goal was (i) to provide a better understanding of the interactions of CO_2_-PMMA, which can also potentially inform the use of CO_2_ on other plastics, and (ii) to highlight experimental conditions that could be safely used for designing conservation strategies, such as cleaning.

## 2. Materials and Methods

### 2.1. Samples

A colorless and transparent PMMA sheet 3 mm thick, produced by cell casting, was bought from PLEXIGLAS^®^ (PLEXIGLAS^®^ GS Clear 0F00 GT, Röhm GmbH & Co., Darmstadt, Germany) supplied with a protective film on both sides. Individual samples measuring approximately 15 mm × 20 mm × 3 mm were cut manually using an electric bandsaw, using the protective film to avoid abrasion and soiling during cutting. The film was removed only immediately prior to sample characterization and testing. No further surface treatment was performed (i.e., rinsing). The PMMA sample batch was stored in the dark in laboratory conditions.

### 2.2. Description of the CO_2_ Apparatus and Experimental Conditions

**Figure 1:**
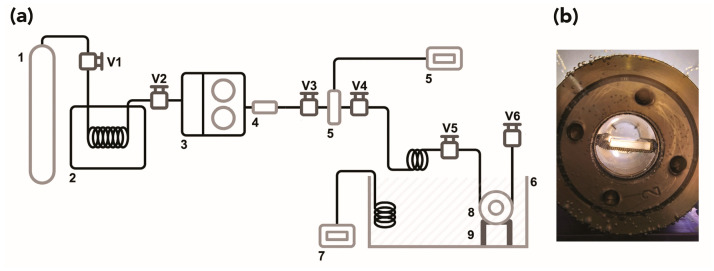
(**a**) Schematic diagram showing the apparatus used for trials: (1) CO_2_ cylinder, (2) refrigeration unit, (3) high-pressure pump, (4) check valve, (5) pressure transducer, (6) thermostatic bath, (7) temperature controller, (8) high-pressure cell with sapphire windows, (9) cell support, (V1) to (V6) high-pressure valves. (**b**) Poly(methyl methacrylate) (PMMA) samples on a stainless-steel grid support inserted in the stainless-steel cell.

Trials were performed in a high-pressure, laboratory-scale apparatus shown schematically in Figure 1a. Experimental conditions (temperature, pressure, exposure, and depressurization time) are summarized in Table 1.

Three specimens (15 mm × 20 mm × 3 mm) per trial were placed on a stainless-steel grid support and then inserted into a 33 mL stainless-steel cell equipped with a sapphire window at each end, which enabled visual access to the samples during the process (Figure 1b). The cell was sealed and immersed in a thermostatic water bath, preheated at the desired temperature. A BlueShadow Pump 40P (Knauer, Berlin, Germany) was used to introduce fresh CO_2_ with 99.998% purity (Air Liquide, Paris, France) until the desired pressure was reached inside the cell. A stream of CO_2_ was then allowed to flow through the vessel for the selected time (Table 1). At the end of each experiment, the cell was manually depressurized at a constant rate to avoid inducing damage to the substrate.

After exposure to CO_2_, samples were left at ambient conditions for at least 2 h before commencing the post-treatment characterization and were subsequently stored in a partially open sample holder under ambient laboratory conditions to allow degassing.

### 2.3. Sample Characterization

#### 2.3.1. Change in Mass

To monitor weight changes (%) and potential physical changes, samples were weighed using a Sartorius CP225D micro analytical balance (Göttingen, Germany) with an accuracy of ±0.00001 g. The humidity inside the balance enclosure was controlled using silica gel. Samples were weighed before and after the test (approximately 2 h), and then again after 2 days, and 1, 2, 4, and 35 weeks. Three independent measurements per sample were taken; the average and the standard deviations were calculated.

#### 2.3.2. Change in Dimensions

Samples’ dimensions (length, width, and thickness) were measured using a TOPEX 31C629 micrometer screw gauge (Grupatopex, Warsaw, Poland), with a length of 135 mm, a 0–25 mm working range, and an accuracy of ±0.01 mm. Samples were measured before, after the test (approximately 2 h), and over time (i.e., after 1, 2, 4, and 35 weeks). Three independent measurements per sample were taken. Average dimensions, volume, and standard deviations were calculated.

#### 2.3.3. Imaging

Full-scale images of the samples before and after tests were acquired with a Dino-Lite^®^ Edge AM7915MZTL microscope (AnMo Electronics Corporation, Taipei, Taiwan) with an Open Cap N3C-O (9.5 mm length), varying Dino-Lite^®^ lighting levels (Flexible LED Control) that were controlled through the DinoCapture 2.0 software (Almere, The Netherlands), at a magnification of approximately ×20 (scale is 2 mm).

Detailed images of the samples’ surfaces were acquired using an Axioplan 2ie microscope (Zeiss, Germany), equipped with an incident halogen light illuminator (tungsten light source, HAL 100) and coupled with a DXM1200F digital camera and ACT-1 control software (Nikon, Japan). Micrographs were captured within a few days of exposure to CO_2_ and again after 35 weeks, using reflected (incident) light in brightfield, darkfield, and cross-polarized modes, and transmitted light in cross-polarized mode, at varied magnifications (×50, ×100, ×200, and ×500).

#### 2.3.4. Raman Spectroscopy (μ-Raman)

Raman microscopy was carried out using a Horiba Jobin Yvon LabRAM 300 spectrometer (Kyoto, Japan), equipped with a He-Ne 17 mW laser operating at 632.8 nm and coupled to the confocal microscope with high-stability Olympus BX41. The system was calibrated using a silicon standard. The laser power at the surface of the samples was reduced with the aid of neutral density filters (optical densities 0.3), and the laser beam was focused with an Olympus ×50 objective. Spectra were recorded as an extended scan, with a grating of 600 groves/mm and an integration time of 10 s. Three spectra for each sample were acquired before and after tests at three independent locations, approximately always the same by using a template mask. Raman data analysis was performed using the LabSpec 5 software. All spectra are presented as acquired, without any baseline correction. The intensities of all spectra were normalized by the peak intensity of the CC4 symmetric stretching mode of PMMA at 813 cm^−1^, following the procedure used by Ikeda-Fukazawa et al. [64]. In this study of the sorption and diffusivity of CO_2_ into PMMA, they noticed that the vibration energy of the CC4 symmetric stretching mode remained almost constant during the CO_2_ sorption, which indicates that this stretching mode is not affected by the sorption process.

#### 2.3.5. Attenuated Total Reflection Fourier Transform Infrared (ATR-FTIR) Spectroscopy

Infrared spectra were obtained using a Handheld Agilent 4300 spectrometer (Agilent, Santa Clara, CA, USA) equipped with a ZnSe beam splitter, a Michelson interferometer, and a thermoelectrically cooled DTGS detector. Spectra were collected with a diamond ATR crystal element, 128 scans and a resolution of 4 cm^−1^, in the spectral region of 4000–650 cm^−1^. Background spectra were collected between every acquisition. Three spectra for each sample were acquired before and after tests and after 2 h, 5 days, and 1, 2, and 35 weeks at three different locations. The OriginPro 8 software (OriginLab Corporation, Northampton, MA, USA) was used to analyze the spectra. All spectra are presented as acquired, without baseline corrections or other treatments except normalization to the carbonyl peak intensity, allowing a direct comparison of relative intensities.

#### 2.3.6. Surface Hardness

Surface hardness was measured with a Zwick/Roell Indentec ZHµ hardness (Gravimeta, Oporto, Portugal) testing machine using a 300 gf load and a dwell time of 15 s. Analysis conditions were selected based on a recent study on the characterization and long-term stability of historical PMMA sheets [9]. Tests were performed on one sample out of the three exposed to each CO_2_ trial. Hardness values and standard deviation were determined as the average of 10 independent readings (5 on each side) obtained at a distance > 5*d* from each other. Measurements were collected approximately 3 h after exposure to CO_2_ and after 2 days and 1, 2, 4, and 35 weeks. Variations in hardness values for each sample over time were determined by comparison with a set of four control samples that had not been exposed to CO_2_.

Hardness values for control and CO_2_-exposed samples were compared using a one-way ANOVA statistical test. Where results were statistically different, a post hoc test (Tukey–Kramer multiple comparison) was performed. Results for these tests are reported above related graphs in lower-case letters. Where no statistical differences were revealed, the bars are labeled with the same letter(s). All statistical tests were conducted using the Origin 2022b software (OriginLab Corporation, Northampton, MA, USA).

## 3. Results and Discussion

### 3.1. Post-Treatment Assessment

#### 3.1.1. Appearance, Weight, Dimensions, and Visual Observations

**Figure 2:**
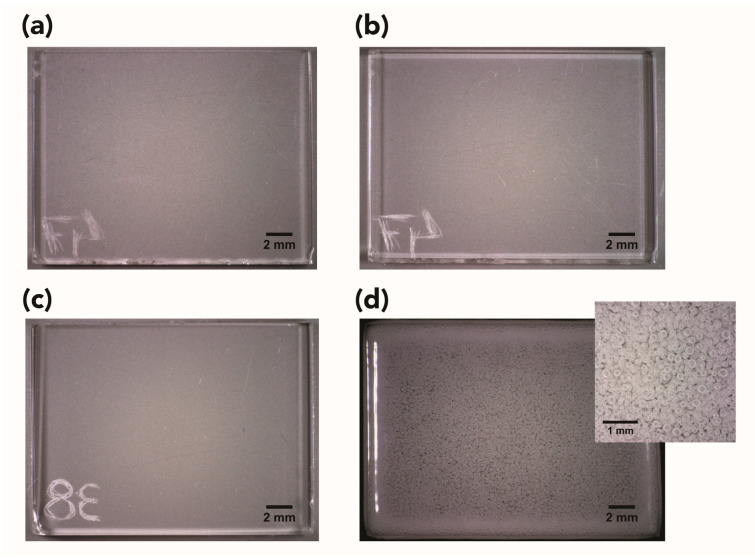
Representative digital photographs for PMMA samples (**a**) before and (**b**) after exposure to liquid CO_2_ at 25 °C and 7 MPa (Test 5) and (**c**) before and (**d**) after exposure to CO_2_ at 55 °C and 28 MPa (Test 4), showing the presence of extensive bubbles trapped in the sample’s bulk.

During the experiment, no visible alterations were detected in the samples by looking through the sapphire windows. However, upon depressurization and removal of the specimens from the high-pressure cell, the samples appeared to have a more glossy surface (judged by the naked eye). Although this effect seemed to reduce within a few days, the appearance of the CO_2_-exposed samples remained (and remains) different compared with the control/unexposed ones. In addition, most of the samples also presented slightly rounded corners, and it was possible to observe some distortions at the edges, which appeared swollen (Figure 2a,b). Significant alterations were noticed for samples subjected to scCO_2_ at 55 °C and 28 MPa (i.e., Test 4), which showed a whitish/milky appearance and the presence of extensive bubbling in the samples’ bulk (Figure 2c,d).

**Figure 3:**
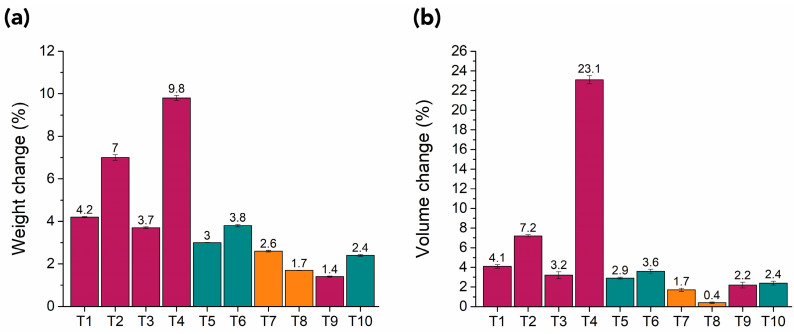
Average and standard deviation values for changes in (**a**) weight and (**b**) volume for PMMA samples treated with CO_2_ at different conditions (supercritical: purple bars; liquid: blue bars; vapor: orange bars), measured approximately 2 h after tests.

Variations in the weight and volume of the samples, measured approximately 2 h after the CO_2_ trials, are summarized in Figure 3. A significant increase in weight was noted for all the samples, with larger changes for specimens treated at 28 MPa (Tests 2 and 4, Figure 2a). Concurrent dilation in thickness, length, and width was also observed, especially for samples exposed to CO_2_ at supercritical conditions and for tests at 28 MPa (Tests 1–4, Figure 3b). Reducing the exposure time to 30 min instead of 60 did not have a significant impact on the response of PMMA to CO_2_ (Test 9 and Test 10, Figure 3a,b); also see Table 1).

Differences in mass and volume expansion indicate sorption/dissolution of CO_2_ into the polymer network, with consequent swelling, as reported in previous studies [65,66,67,68]. Polymers show very low solubility in CO_2_, which is a function of temperature, pressure, and concentration, but also depends on the polymers’ molecular weight (Mw) and molecular weight distribution [69,70,71,72]. According to the literature, CO_2_ is a good solvent for many nonpolar and some polar molecules with low Mw, including most common monomers and oligomers, but has limited solubility for larger components and polymers with Mw above 1000 [69,70,73]. By contrast, CO_2_′s solubility in polymers might be considerable and associated with swelling of the matrix [32,74].

The sorption and swelling behavior of PMMA/CO_2_ systems have been widely studied, mainly via in situ experiments (i.e., while the specimens are inside the CO_2_ apparatus), exploring a wide range of temperature and pressure conditions, and through different analytical methods [75,76,77,78,79,80,81,82,83,84,85,86,87,88,89,90,91,92,93].

The dissolution of carbon dioxide in a polymer matrix is driven by various factors: temperature and pressure experimental conditions (sorption is greater at higher pressure and relatively low temperatures), polymer morphology and degree of crystallinity, and interaction between CO_2_ and specific functional groups in the polymer (such as carbonyl groups or phenyl rings) [94,95]. Glassy polymers, particularly PMMA, have stronger CO_2_ solubility than semicrystalline/crystalline polymers and exhibit larger weight variations due to CO_2_ uptake [65,66,68,80]. As a highly amorphous polymer, PMMA has little molecular orientation and large free volume, whereas crystalline polymers have highly ordered molecular arrangement and relatively less free volume; hence, CO_2_ is not absorbed as easily by them. Weight and volume variations shown in Figure 3 are in line with observations from previous studies, with bigger changes noted for tests performed at high pressure (i.e., 28 MPa).

Sorption of CO_2_ into a polymer matrix is also reported to promote plasticization and reduction of the glass transition temperature (*T*_g_) [96,97,98,99,100], as well as the formation of a cellular/porous structure [67,87,101,102,103]. The formation of bubbles might occur during the depressurization stage, and bubbles are more likely to form when the operational conditions are of high temperature and/or high pressure and the depressurization to ambient conditions is performed very quickly [104]. Carbon dioxide impregnates the polymer matrix to a different degree, depending on various factors, such as experimental conditions and polymer type [74]. Upon depressurization, CO_2_ that has already dissolved in the polymer matrix can become supersaturated and nucleate bubbles, which induces foam or minor defects in the polymer structure [105,106,107,108]. Induced bubble formation, growth, and foaming are methods widely used in polymer processing for various applications, such as creating a porous structure into polymers, and for drug loading [32,73,74,109,110].

**Figure 4:**
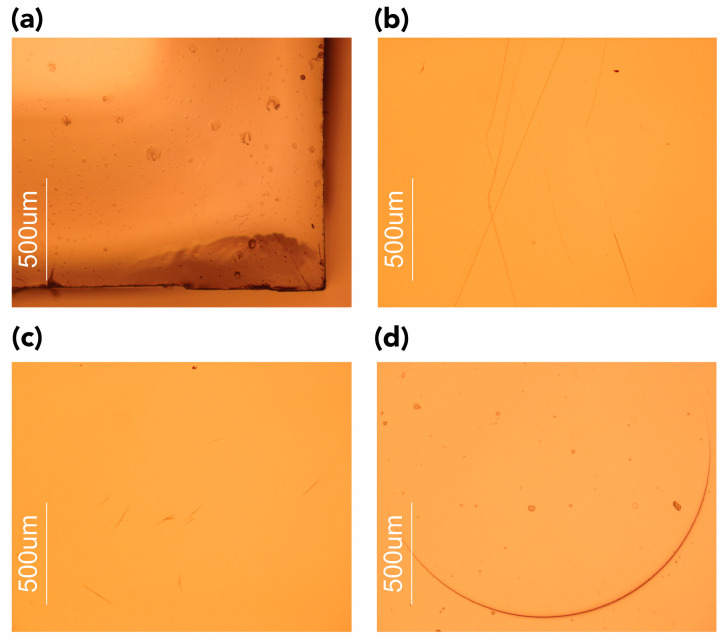
Representative microphotographs acquired in reflected light in brightfield showing (**a**) the presence of abrasion and (**b**) cracks, (**c**) free and impinged bubbles in the sample’s bulk, and (**d**) indentation mark left by the tip of the pressure clamp used to ensure contact between the exposed test sample and the crystal during ATR-FTIR spectroscopy. Magnification ×50.

In the present study, cavities/bubbles were readably visible in samples subjected to scCO_2_ at 55 °C and 28 MPa (i.e., Test 4, Figure 2c,d). Tiny, discrete bubbles invisible to the naked eye were noticed in samples exposed under experimental conditions of 35 °C and 28 MPa (Figure 4a) when examined with optical microscopy (OM). For all the other tests, no cavities/bubbles were observed, and this could be due to the mild experimental conditions used and the relatively slow depressurization rate.

Optical microscopy examination also highlighted the presence of other small defects and physical damage. Regardless of the experimental conditions used, surface scratches, potential crazing, and small cracks were observed (Figure 4b,c).

In addition, one could observe the presence of indentation marks left by the tip of the pressure clamp used in ATR-FTIR spectroscopy (Figure 4d). These marks were not observed on control samples.

**Figure 5:**
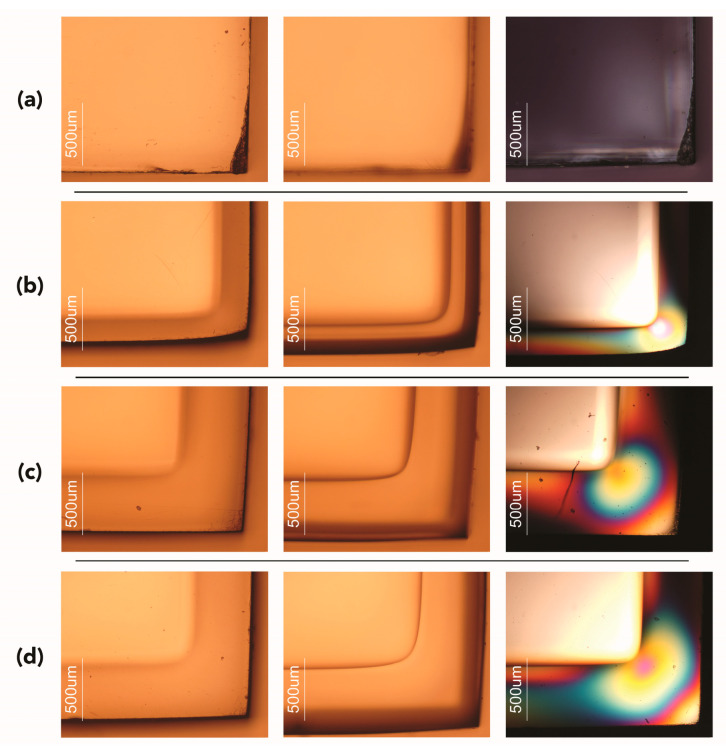
Representative microphotographs in brightfield (left and middle columns) and cross-polarized light (right column) showing the optical boundary and the isochromatic fringes for (**a**) control untreated sample, (**b**) sample exposed to vapor CO_2_ at 25 °C and 6 MPa, (**c**) sample exposed to liquid CO_2_ at 25 °C and 10 MPa, (**d**) sample exposed to supercritical CO_2_ at 35 °C and 10 MPa. Note: images in the left column were focused on the surface, while images in the middle column were focused inside the sample to better visualize the optical boundary, and hence, the surface appears slightly unfocused. Magnification ×50.

Analysis of the distorted and swollen areas showed the presence of a continuous, solid line or optical boundary inside the samples in the proximity of the edges (Figure 5, left and middle columns). The formation of the optical boundary can be observed in optically transparent polymers and is associated with the sorption and diffusion of a solvent through the samples. In previous studies, the analysis of the propagation front via optical microscopy was used to study in situ the diffusion of methanol in PMMA and dodecane in polystyrene (PS) [111] or the swelling and sorption kinetics of scCO_2_ in poly(dimethylsiloxane) [112], PS [113], PMMA, and poly(butyl methacrylate) (PBMA) [67,81,82]. These studies showed that the boundary appears after a few minutes of exposure to high-pressure CO_2_ and propagates slowly in all directions until it contracts and disappears in the center when the phase equilibrium between the CO_2_ and the polymer has been reached; that is, the CO_2_ is completely absorbed by the specimen.

In the current study, the experiments were stopped before reaching the equilibrium phase, and the assessment was performed ex situ. It was therefore possible to still observe the optical boundary when the samples were removed from the high-pressure cell. The propagation of the optical front is consistent with data collected for weight changes and swelling. From a visual assessment, its size seems dependent on the experimental conditions: the more intense the conditions, the more the CO_2_ penetrates into the PMMA; hence, the bigger the optical boundary. Further analysis of the optical boundary and its correlations with the experimental conditions used in this study were evaluated via molecular dynamics studies and will be presented in a forthcoming paper.

For the PMMA/CO_2_ binary system, the presence of the optical boundary not only indicates CO_2_ uptake but also represents an interface between glassy and plasticized regions, as recently discussed by Rodríguez et al. [114].

Compared with control samples (Figure 5a), the CO_2_-exposed samples also showed differences in appearance and color in the areas defined by the optical boundary when observed under polarized light using an optical microscope (Figure 5b–d). In all mock-ups, the presence of distinct isochromatic fringes corresponding to the optical boundary indicated stress-induced regions (Figure 5, right column). The different colors correspond to different stress levels, and the higher the density of the color fringes, the greater the stress [115,116].

#### 3.1.2. Mechanical Properties

**Figure 6:**
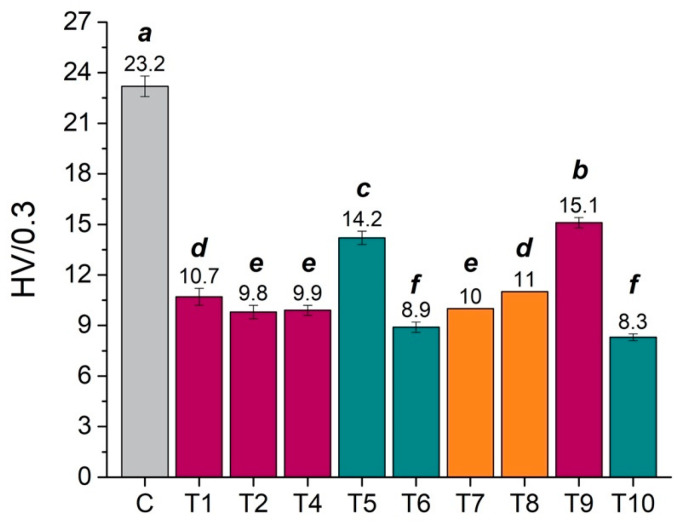
Vickers hardness values (average and standard deviations) for a control, unexposed sample (gray bar labeled C) and CO_2_-exposed samples (colored bars T1–T10) at different conditions (supercritical: purple bars; liquid: blue bars; vapor: orange bars), measured approximately 4 h after tests. Statistical significance for ANOVA and Tukey–Kramer multiple comparison tests was established at a *p*-value < 0.05; mean values that do not share a superscribed letter are significantly different.

A significant decrease in the surface hardness of CO_2_-exposed samples compared with a set of controls was registered a few hours after tests. Corresponding Vickers hardness values (HV) are summarized in Figure 6. Lower HV values are attributed to a plasticization effect [96,97,98,99,100] and reduction of the glass transition temperature (*T*_g_) due to the sorption of CO_2_ into the amorphous, unstructured regions in PMMA, as previously discussed. These conditions can promote localized rearrangements in pockets of free volume within the polymer network. The consequence is a significant increase in the chain mobility and intermolecular distances between them, which induce disentanglement and reorientation of the chains to a more thermodynamically favorable “crystalline” state [65,66,80,90,97,117,118,119]. These effects indicate that the CO_2_-exposed polymer is significantly plasticized by carbon dioxide with the potential of changing the mechanical properties of the material [65,66].

An indirect indication that the softening of the surface had occurred was also provided using optical microscopy (see Figure 4). After tests, the surface of all samples presented several scratches, abrasion scuffs, and marks produced while running ATR-FTIR spectra, suggesting that the samples were more fragile and prone to damage than before treatment. Special care and caution should be taken when removing the samples from the high-pressure cell and when handling and analyzing them.

Surface softening occurred for all specimens, and corresponding micro-hardness values were within the same range. However, the ANOVA post hoc Tukey–Kramer multiple comparison tests highlighted significant differences among the various CO_2_ trials, as shown in Figure 6 (mean values that do not share a letter are significantly different). It should be noted that hardness measurements for Tests 5 and 9 (see Table 1) were performed approximately 15 h after tests, rather than 4 h as for all the other samples, and this might explain the higher HV value.

Changes in the mechanical properties of PMMA (and other polymers) samples subjected to exposure to CO_2_ under different conditions were also registered by other authors with the aid of different techniques. That is, a decrease in tensile strength and Young’s modulus was also observed [65,66,68].

#### 3.1.3. Spectroscopic Examinations

**Figure 7:**
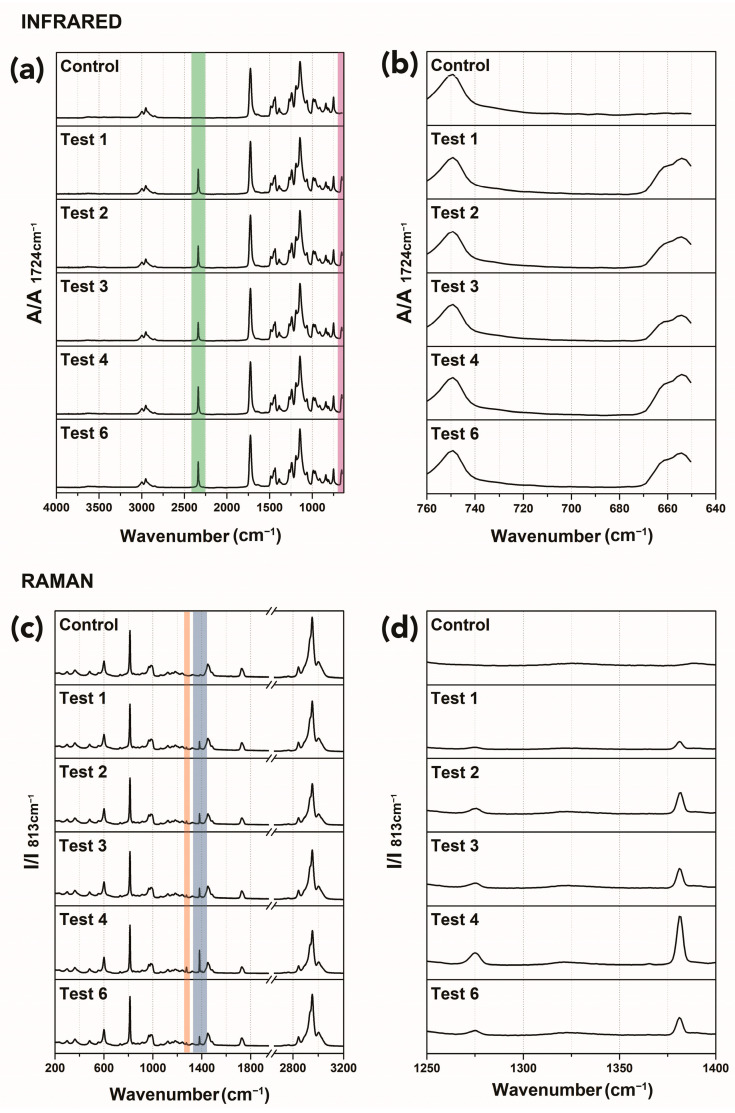
(**a**) ATR-FTIR spectra overlaid, (**b**) detail of the spectra in the region 760–640 cm^−1^, (**c**) Raman extended spectra, and (**d**) detail of the Raman spectra in the region 1250–1400 cm^−1^, for control (untreated PMMA sample) and samples subjected to CO_2_ at different conditions. The colored rectangles in the spectra highlight the CO_2_ absorption bands at approximately 2338 cm^−1^ (highlighted in green, panel a) and at 662 and 654 cm^−1^ (highlighted in violet, panel a), while the orange and blue rectangles in the Raman spectra (panel c) highlight the peaks at 1391 and 1286 cm^−1^, respectively. All peaks are attributed to the CO_2_ in the gas phase and dissolved in PMMA.

Figure 7 shows ATR-FTIR spectra (top, panels (a) and (b)) and Raman spectra (bottom, panels (c) and (d)) for control and samples exposed to CO_2_ at different conditions (Tests 1–4 and Test 6; see Table 1).

All spectra present a similar profile, which is representative of the PMMA homopolymer. The ATR-FTIR spectrum of the control sample (Figure 7, panels (a) and (b)) shows the following diagnostic peaks: C–H stretching (at 2995, 2951, and 2843 cm^−1^), C–C–O stretching (at 1269 and 1239 cm^−1^), C=O carbonyl stretching absorption peak (1731 cm^−1^), C–O–C stretch (1190 and 1143 cm^−1^) [120]. After trials, new absorption bands developed that were attributed to CO_2_ sequestered within the polymer, namely, a band at approximately 2338 cm^−1^ (highlighted in green in Figure 7a) and at 662 and 654 cm^−1^ (highlighted in violet in Figure 7a), the latter clearly visible in Figure 7b, where a detail of the 760–640 cm^–1^ region is shown [94,95,121].

Typical vibration bands for PMMA as analyzed by Raman spectroscopy (Figure 7c, control) are the C–H stretching vibration peaks (~2996, 2949, and 2842 cm^−1^), carbonyl stretching (1727 cm^−1^), and C–H bend (at 1450 cm^−1^), according to the literature [122,123]. Spectra for samples exposed to CO_2_ show the presence of additional sharp peaks that can be attributed to CO_2_ in the gas phase and dissolved in PMMA [64,124], namely, the peaks at 1391 and 1286 cm^−1^, highlighted in blue and orange, respectively, in Figure 7c and more clearly visible in Figure 7d, where a detail of the region 1250–1400 cm^−1^ is shown.

### 3.2. Long-Term Assessment

The loss of absorbed carbon dioxide from the polymer was monitored through mass reduction with time [80].

**Figure 8:**
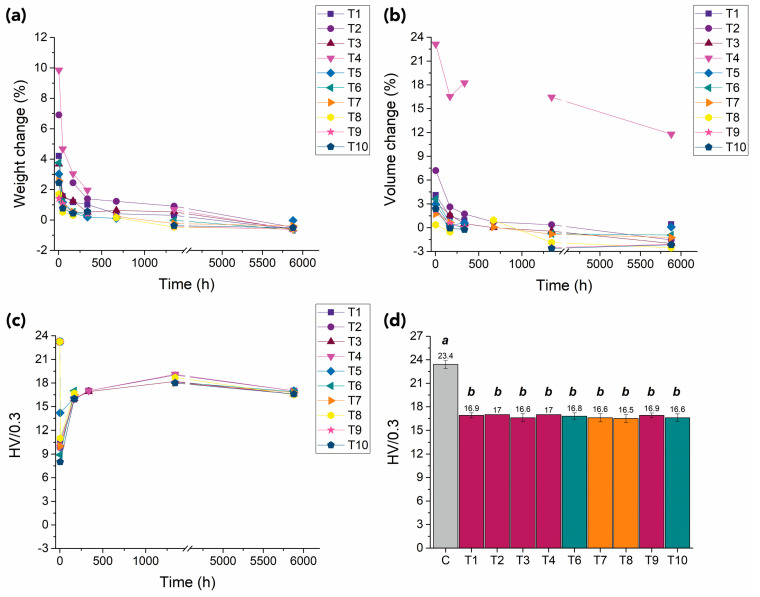
Summary of changes over time (up to 35 weeks, i.e., ~6000 h) in (**a**) weight, (**b**) volume, and (**c**) mean Vickers hardness values for PMMA samples exposed to CO_2_ under different conditions. (**d**) Vickers hardness values (average and standard deviation) after 35 weeks for a control, an untreated sample (gray bar labeled C), and CO_2_-exposed samples (colored bars T1–T10) at different conditions (supercritical: purple bars; liquid: blue bars; vapor: orange bars). Statistical significance for ANOVA and Tukey–Kramer multiple comparison tests was established at a *p*-value < 0.05. Mean values that do not share a letter are significantly different and vice versa.

The desorption of CO_2_ was similar for all exposed samples (Figure 8a). Degassing occurred quickly within a few days and then slowed exponentially with time, with specimens reaching their original weight values. Within approximately 6000 h (~35 weeks), all samples showed maximum weight loss. Alterations were, however, in the range of 0.6% of the original weight, considerably below what could be attributed to experimental error; hence, these changes are not significant. More significant alterations in polymers’ weight after exposure to CO_2_ might be attributed to the extraction of monomers, oligomers, additives, stabilizers, processing aids, and plasticizers, as reported in the literature [88,89]. The samples’ dimensions also recovered to original values except for samples treated at 55 °C and 28 MPa (Test 4), which remained visibly swollen (Figure 8b).

With the desorption of CO_2_ from the samples, an increase in the hardness value was observed (Figure 8c). This can be related to a further rearrangement of the polymer chains occurring while the CO_2_ leaves the samples. Hardness values stabilized approximately 20 days after tests (~480 h). Oscillations in the values might be related to humidity uptake [9]. After 35 weeks, HV values were similar for all samples, with no significant differences noted by the ANOVA post hoc Tukey–Kramer multiple comparison tests (Figure 8d). Even though recovery of the hardness occurs with time, the values of the CO_2_-exposed samples remained lower and significantly different from the initial ones for the control of unexposed PMMA.

**Figure 9:**
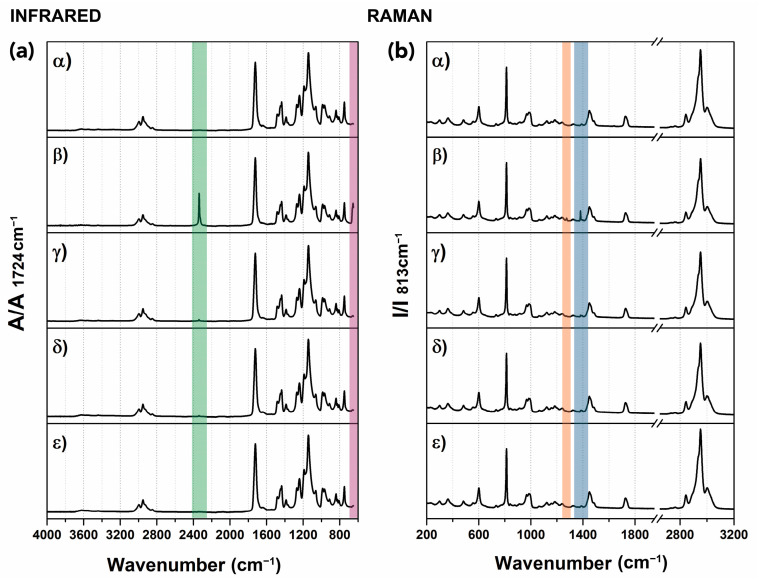
ATR-FTIR and Raman spectra for a sample exposed to scCO_2_ at 35 °C and 10 MPa (**α**) before the test (**β**), 2 h after the test (**γ**), 5 days after the test (**δ**), 1 week after the test (**ε**), and 35 weeks after the test. The colored rectangles in the infrared graph highlight the CO_2_ absorption bands at approximately 2338 cm^−1^ (highlighted in green) and at 662 and 654 cm^−1^ (highlighted in violet), while the orange and blue rectangles in the Raman spectra highlight the peaks at 1391 and 1286 cm^−1^, respectively. All peaks are attributed to the CO_2_ in the gas phase and dissolved in PMMA. CO_2_ peaks disappeared 5 days after exposure.

After 35 weeks, ATR-FTIR and Raman spectra of the CO_2_-exposed samples remained unaltered and similar to the control profile, suggesting that no molecular alterations had occurred due to exposure. Peaks assigned to CO_2_ almost completely disappeared from both infrared and Raman spectra 5 days after trials. A representative example is reported for Test 1 (scCO_2_ at 25 °C and 7 MPa) in Figure 9.

**Figure 10:**
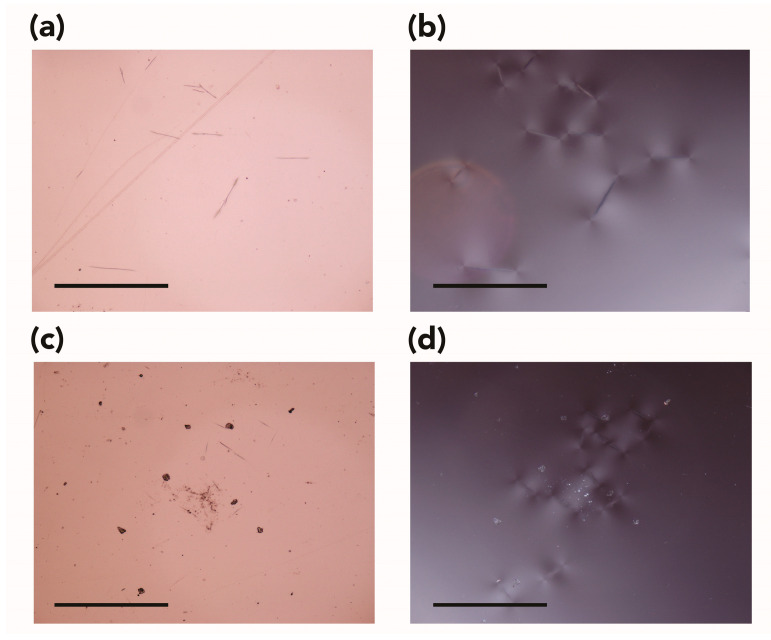
Representative microphotographs for: (**a**) control untreated sample, in reflected light in brightfield; (**b**) sample exposed to vapor CO_2_ at 25 °C and 6 MPa, in transmitted cross-polarized light; (**c**) sample exposed to liquid CO_2_ at 25 °C and 10 MPa, in reflected light in brightfield; (**d**) sample exposed to supercritical CO_2_ at 35 °C and 10 MPa, transmitted cross-polarized light. Magnification ×50.

The visual appearance of the samples dramatically changed. Specimens became slightly shinier (assessed by the naked eye), and a dense network of fractures and stress-induced cracking became more evident over time, completely covering the samples’ surfaces (Figure 10).

## 4. Conclusions

This paper presented a systematic study on the effects of carbon dioxide at different conditions (supercritical, liquid, and vapor) on a new poly(methyl methacrylate) cast sheet to evaluate its safety and suitability as a solvent for conservation purposes. A multi-analytical approach involving OM under reflected and transmitted light (in brightfield and cross-polarized light modes, respectively), μ-Raman and ATR-FTIR spectroscopies, and micro-indentation was adopted to capture and follow potential changes in the appearance and physical, chemical and mechanical properties of the samples. The data collected showed that liquid and supercritical CO_2_ strongly interacted with the PMMA samples, inducing irreversible changes. Similar effects were also observed for tests performed with vapor CO_2_.

The most dramatic alterations noted involved changes in the visual aesthetic of the samples and their mechanical properties. Following exposure to CO_2_, a drastic decrease in the surface hardness of the samples was noted. The exposed specimens became more fragile and prone to scratching, which warrants caution when handling the samples. Observation under OM revealed the presence of severe scuffs and marks, including indentation marks left during ATR spectroscopy, which were not observed in samples not subjected to CO_2_. Smaller alterations were recorded if samples were exposed to CO_2_ for a shorter period (i.e., 30 min, Tests 9 and 10). Modification to the appearance of the samples occurred slowly and over time. A shift in the specimens’ gloss and some distortions around the edges were noticed immediately after trials. While these effects diminished slightly with time, a dense network of fractures and stress-induced cracking developed, covering the samples’ surfaces completely.

Considering that the modifications observed have compromised the samples’ aesthetic and future stability, it can be stated that CO_2_ is unsuitable as a green solvent for conservation treatments of PMMA. Polymer-based objects in cultural heritage collections exhibit different formulations and intrinsic characteristics; hence, the modifications observed on PMMA should not be expected to occur on all other plastics if exposed to CO_2_. For example, amorphous polymers might experience severe sorption/dissolution of carbon dioxide into the polymer network, with consequent swelling, dilation, extraction of plasticizers or other additives, and modification of the mechanical properties. In contrast, crystalline polymers, polyurethanes, and other foams might be less affected. Further research on the use of CO_2_ as a solvent for the treatment of other synthetic polymers will be the focus of forthcoming publications.

## Figures and Tables

**Table 1:** Summary of experimental conditions used for CO_2_ trials on poly(methyl methacrylate) (PMMA).

Test No.	Temperature (°C)	Pressure (MPa)	CO_2_ Density (g/mL)	CO_2_ Phase	Flux (mL/min)	Compression Time (min)	Exposure Time (min)	Depressurization Time (min)
1	35	10	0.71281	Supercritical	10	~15	60	20
2	35	28	0.91864	Supercritical	10	~15	60	20
3	55	10	0.32507	Supercritical	10	~15	60	20
4	55	28	0.8357	Supercritical	10	~15	60	20
5	25	7	0.74303	Liquid	10	~15	60	20
6	25	10	0.81763	Liquid	10	~15	60	20
7	35	6.7	0.19798	Vapor	10	~15	60	20
8	25	6	0.19061	Vapor	10	~15	60	20
9	35	10	0.71281	Supercritical	10	~15	30	20
10	25	10	0.81763	Liquid	10	~15	30	20

## Data Availability

Not applicable.
